# 16S rRNA sequencing-based evaluation of the protective effects of key gut microbiota on inhaled allergen-induced allergic rhinitis

**DOI:** 10.3389/fmicb.2024.1497262

**Published:** 2025-01-09

**Authors:** Yi Tang, Yongchuan She, Danping Chen, Yibo Zhou, Dan Xie, Zhai Liu

**Affiliations:** Changsha Hospital of Traditional Chinese Medicine (Changsha Eighth Hospital), Changsha, China

**Keywords:** allergic rhinitis, 16S rRNA sequencing, diagnosis, fresh feces, microbiota

## Abstract

**Introduction:**

Allergic rhinitis (AR) is a common respiratory disorder influenced by various factors in its pathogenesis. Recent studies have begun to emphasize the significant role of gut microbiota in immune modulation and its potential association with the development of AR. This research aims to characterize the gut microbiota of patients with AR who are sensitized via inhalation, utilizing 16S rRNA sequencing to shed light on the pathogenesis of AR and identify potential therapeutic targets.

**Methods:**

To achieve the study’s objectives, we compared the microbiota profiles between patients with AR and healthy controls. Microbial diversity was assessed using alpha and beta diversity indices, and differential microbiota populations were identified through Linear discriminant analysis Effect Size (LEfSe) analysis. A Least Absolute Shrinkage and Selection Operator (LASSO) regression model was employed to pinpoint key species. Additionally, PICRUSt2 was utilized to predict the functional pathways associated with these identified species.

**Results:**

The analysis identified a total of 1,122 common species, along with 1,803 species associated with AR and 1,739 species associated with healthy controls. LEfSe analysis revealed 20 significant discrepancies at the genus level. The LASSO regression model identified 8 key genera, including *Prevotellaceae* UCG-004 and *Rhodococcus*, which exhibited AUC values exceeding 0.7, indicating strong diagnostic potential. Furthermore, functional pathway analysis suggested that these pivotal species are involved in pathways such as L-lysine biosynthesis and photorespiration, potentially contributing to the pathogenesis of AR.

**Discussion:**

This study identifies critical gut microbiota that could serve as potential biomarkers for allergic rhinitis, providing new insights into its pathogenesis and offering avenues for future therapeutic strategies. Further investigation into these microbiota may lead to enhanced understanding and management of AR.

## 1 Introduction

Allergic rhinitis (AR) is a prevalent chronic inflammatory disorder of the respiratory tract, clinically characterized by rhinorrhea, sneezing, nasal congestion, obstruction, and itching (Li S. et al., 2022; Meng et al., 2020). The etiology of AR is multifactorial, shaped by both genetic predisposition and environmental factors. AR often coexists with bronchial asthma, allergic conjunctivitis, and chronic rhinosinusitis, severely affecting patients’ quality of life and imposing a significant economic burden. Globally, AR impacts approximately 5–50% of the population (Wise et al., 2023). Current treatments primarily include nasal corticosteroids, nasal and oral H1-antihistamines, anticholinergics, antileukotrienes, α-mimetic agents, and chromones. However, these therapies frequently exhibit limited efficacy and are associated with a range of side effects (Bousquet et al., 2020b). Prolonged use of allergy medications may result in adverse effects such as somnolence, gastrointestinal disturbances, xerostomia, vertigo, cephalalgia, and increased susceptibility to infections (Makino et al., 2019). Additionally, the therapeutic efficacy of these drugs is often constrained by the timing of administration relative to allergy onset. Consequently, developing a safe, long-term preventive approach for AR is of critical importance (Steiner and Lorentz, 2021). Previous research has demonstrated that the microbiome directly influences inflammatory responses in allergic diseases, including AR and asthma (Schaefer and Enck, 2019; Hirata and Kunisawa, 2017; Pascal et al., 2018). Therefore, understanding the microbiota composition is essential for elucidating AR pathogenesis and identifying potential therapeutic strategies.

Recent studies underscore the pivotal role of diverse gut microbiota in maintaining immune homeostasis and promoting health through the production of metabolites, such as anti-inflammatory short-chain fatty acids, while concurrently reducing inflammatory mediators such as lipopolysaccharides (Richards et al., 2016; Laitinen and Mokkala, 2019). Microbial colonization during prenatal, neonatal and early childhood stages shapes the gut environment and profoundly impacts immune system development. Disturbances in microbiota colonization during these critical periods may compromise the future immune function and increase susceptibility to allergic conditions (Kaczynska et al., 2022). AR, a common upper respiratory tract disease, significantly impacts patients’ quality of life, particularly during frequent episodes (Tierney et al., 2019). The gut microbiota contains over 1,500 bacterial species, with certain species being ubiquitous across healthy individuals. The phyla Firmicutes, Bacteroidetes, Actinobacteria, and Proteobacteria account for approximately 90% of the total microbial population (Wright et al., 2015). Previous studies have shown that altered gut microbiota diversity is more prevalent in individuals with allergic diseases. However, the microbial composition varies across different types of allergies, and not all allergic conditions are associated with the same bacterial species (Kaczynska et al., 2022). Despite this, clinically applicable biomarkers to predict AR subtypes, disease severity, and the development of comorbidities remain scarce (Bousquet et al., 2020a). Therefore, identifying reliable biomarkers for AR is of paramount importance.

16S ribosomal RNA (rRNA) sequencing has become a cornerstone in microbiome research, enabling rapid and precise identification, classification, and detection of pathogens. In this study, 16S rRNA sequencing was employed to characterize the dominant gut microbiota in fecal samples from patients with AR sensitized by inhaled allergens. The study also assessed the diagnostic potential of distinctive microbiota at the genus level for AR and investigated the associated metabolic pathways to elucidate their potential roles in AR pathogenesis. This study aimed to identify microbiome differences in patients with AR, providing novel insights into AR diagnosis and potential therapeutic targets.

## 2 Materials and methods

### 2.1 Specimen acquisition

Patients with AR were recruited based on clinical criteria, including a history of allergic symptoms and a positive skin prick test. Healthy controls (HC) were age- and sex-matched individuals without any history of allergic diseases. Exclusion criteria for both groups included recent antibiotic use, probiotic supplementation, or gastrointestinal disorders. Fresh fecal samples were collected in sterile containers either in patients’ homes or in a clinical setting within 24 h of their last meal. The samples were immediately stored on ice and transported to the laboratory within 2 h, where they were preserved at −80°C until DNA extraction. Written informed consent was obtained from all participants prior to sample collection, and the study was approved by the Institutional Review Board of the Medical Ethics Committee of Changsha Hospital of Traditional Chinese Medicine (Changsha Eighth Hospital) (Approval No. 2022031).

### 2.2 DNA extraction

According to the manufacturer’s instructions, microbial DNA was extracted from 200 mg of each fecal sample using the Magnetic Bead Fecal Genome DNA Extraction Kit (Bayer, Leverkusen, North Rhine Westphalia, Germany). Briefly, samples were lysed in a bead-containing lysis buffer, followed by mechanical disruption using a bead beater at (specific speed) for (duration). After lysis, DNA was bound to magnetic beads, washed multiple times to remove contaminants, and then eluted in (volume) of nuclease-free water. The purity and concentration of the extracted DNA were assessed using a NanoDrop spectrophotometer NanoDrop spectrophotometer (Thermo Fisher Scientific, Waltham, MA, United States), with an A260/A280 ratio of 1.8–2.0 indicating good purity. DNA integrity was further confirmed by agarose gel electrophoresis, ensuring the presence of high molecular weight DNA. The extracted DNA samples were stored at −20°C until further analysis, with aliquots prepared for long-term storage to minimize multiple freeze–thaw cycles. On average, the DNA yield per sample was (specific range) ng/μL, with high extraction efficiency observed across all samples. Sufficient DNA was obtained for downstream 16S rRNA sequencing.

### 2.3 Amplification, purification, and quantification of polymerase chain reaction products

Primers, 341F (5′-CCTACGGGNGGCWGCAG-3′) and 805R (5′-GACTACHVGGGTATCTAATCC-3′), were used to amplify the target regions. Thermal cycling involved an initial denaturation at 98°C for 30 s, followed by 30 cycles of denaturation at 98°C for 10 s, annealing at 54°C for 30 s, and extension at 72°C for 45 s, with a final extension at 72°C for 10 min. Polymerase chain reaction (PCR) products were purified using AMPure XT beads (Beckman Coulter Genomics, Danvers, MA, United States) and quantified using a Qubit fluorometer (Invitrogen, Carlsbad, CA, United States).

### 2.4 Library construction and 16S rRNA sequencing

The purified PCR products were assessed with the Agilent 2100 Bioanalyzer (Agilent, Santa Clara, CA, United States) and the Illumina library quantification kit (Kapa Biosciences, Woburn, MA, United States). Libraries with concentrations exceeding 2 nM were considered qualified. The libraries, meeting the quality criteria and featuring non-repeating index sequences, were gradually diluted and mixed proportionally based on the desired sequencing volume. They were then denatured into single strands using NaOH before sequencing. Sequencing was conducted on the NovaSeq 6000 platform (Illumina) using 2 × 250-bp paired-end sequencing with the NovaSeq 6000 SP Reagent Kit (Illumina) (500 cycles).

### 2.5 Data processing

The raw sequencing data were initially processed through splicing, filtering, and de-chimerization using deficiency of adenosine deaminase 2 DADA2 version 1.26.0 (Callahan et al., 2016) with default parameters. Splicing involved merging paired-end reads based on overlapping regions, followed by quality filtering to remove low-quality sequences and those containing ambiguous bases. De-chimerization was then applied to identify and eliminate chimeric sequences using DADA2.

Filtered sequences were subsequently analyzed to generate amplicon sequence variants (ASVs) using the Mothur algorithm, which differentiates sequences based on unique genetic characteristics. The resulting ASV table, containing the abundance of each ASV across samples, was used for downstream diversity analysis and taxonomic classification. The classification was performed using the (The Max Planck Institute for Marine Microbiology is one of the main maintenance institutions), dated 3 October 2024) database.

### 2.6 Species composition and diversity analyses

Species diversity and relative abundance were assessed by computing rank-abundance curves, rarefaction curves, and species accumulation curves using the rankabund(), the rarecurve(), and specaccum() functions from the R (version 2.6.2) software package. Based on ASV annotations, the relative abundance of gut microbiota in each sample was analyzed at various taxonomic levels (domain, phylum, class, order, family, genus, and species).

To investigate species diversity in intestinal micropopulations among patients with AR α-diversity was estimated using species richness, Abundance-based Coverage Estimmator ACE, Chao1, Shannon, Simpson, and coverage indices. Statistical comparisons between AR and HC were conducted using the statistical function in R (version 4.3.3) software package, with Welch’s *t*-test used to evaluate α-diversity across samples. A *p* < 0.05 was considered statistically significant.

For β-diversity, principal coordinate analysis (PCoA) and analysis of similarities (ANOSIM) were performed using the R (version 2.6.2) software package. Non-metric multidimensional scaling (NMDS) was also applied to explore similarities in bacterial community structures between groups. Linear discriminant analysis (LDA) effect size (LEfSe) was used to identify dominant micropopulations. The Wilcoxon rank–sum test was applied to validate the consistency of differences across subgroups. LDA was further used to estimate the contribution of each species to the differential outcomes. LEfSe (dated 3 October 2024) was used to select significantly different micropopulations between AR and HC, defined as discrepant micropopulations (LDA score >4, *p* < 0.05). Additionally, a collinear network at the genus level was constructed using Spearman correlation analysis to visualize relationships among discrepant micropopulations, with correlations of *R* > 0.8 and *p* < 0.05 considered significant (Yang et al., 2022).

### 2.7 Screening and functional inquiry of pivotal micropopulations

A least absolute shrinkage and selection operator (LASSO) regression model was developed to identify pivotal micropopulations, optimizing for the highest model precision. To evaluate the diagnostic potential of these pivotal micropopulations, the pROC package (version 1.18.4) (Robin et al., 2011) in R was used to generate receiver operating characteristic (ROC) curves, with an area under the curve (AUC) >0.7 considered indicative of strong diagnostic value. To further understand the metabolic functions of these pivotal micropopulations, Phylogenetic Investigation of Communities by Reconstruction of Unobserved States 2 (PICRUSt2) was employed to predict the enriched pathways associated with key micropopulations, with a *p* < 0.05 considered significant.

### 2.8 Statistical analysis

Bioinformatics analyses were performed using R (version 4.3.1) software package (R is a programming language and environment used for statistical computing and graphing, and is jointly developed and maintained by numerous statisticians and programmers). Inter-group differences were assessed using the Wilcoxon rank-sum test and pairwise comparisons were conducted using LEfSe analysis. A *p*-value or adjusted *p*-value (*p*.adj) of less than 0.05 was considered statistically significant.

## 3 Results

### 3.1 Comparison of species composition between AR and HC groups

**Figure 1:**
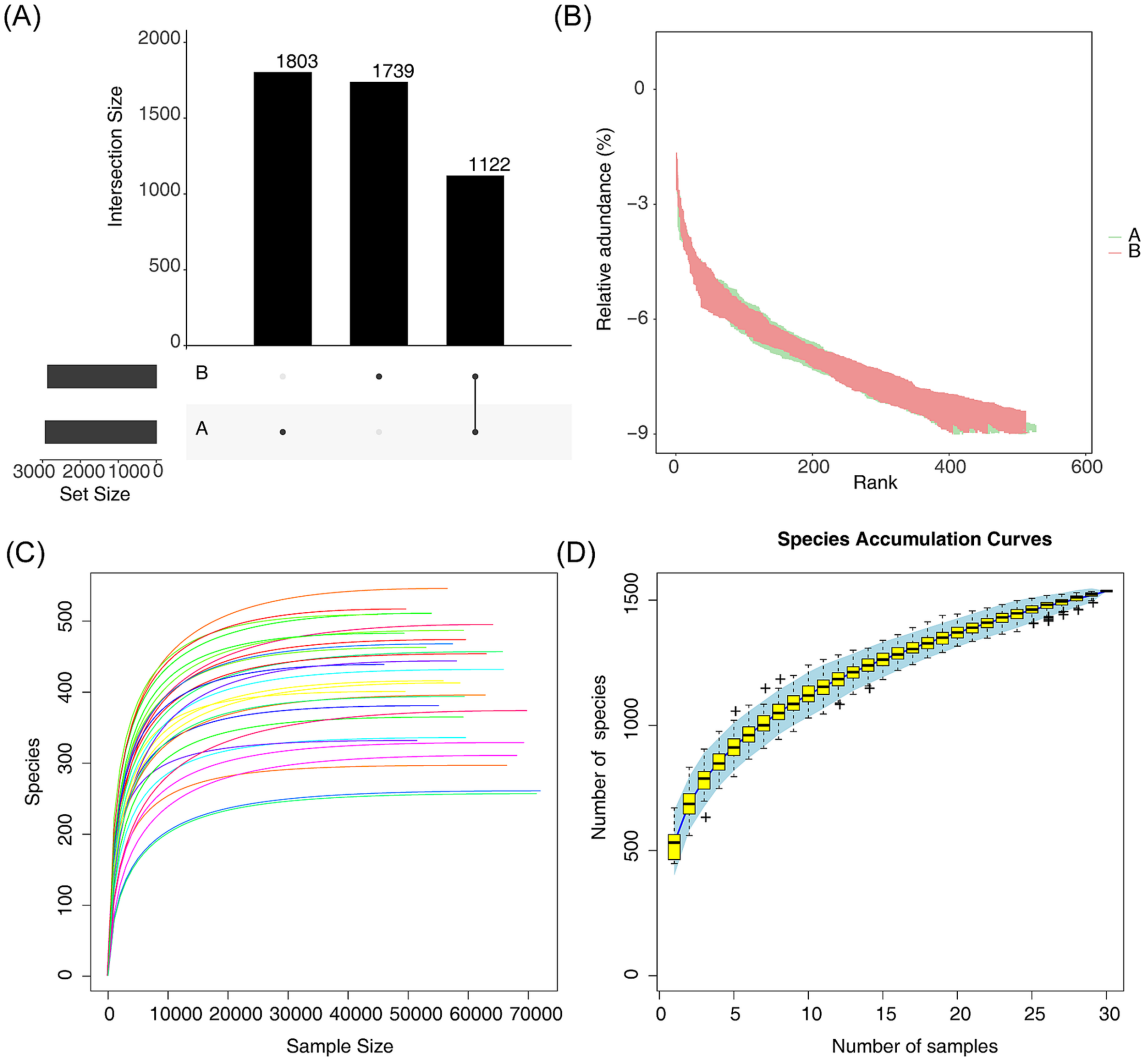
Species composition and diversity analyses between AR and HC. **(A)** Venn diagrams comparing endemic or common microbiota between AR and HC, with A and B denoting endemic species, and black dots indicating the presence of species. **(B)** Rank-abundance curves quantifying species distribution and composition in the community, with endemic species A shown in green and endemic species B in red. **(C)** Rarefaction curves that were generated from species counts of sequencing data were extracted from each sample, with different colored curves representing data from individual samples. **(D)** Box plot analyzing sample size adequacy between AR and HC.

The Venn diagram was employed to analyze both the endemic and common microbiome at the species level between AR and HC. This analysis revealed 1,122 common species, 1,803 AR-endemic species, and 1,739 HC-endemic species (Figure 1A). Rank-abundance curves were used to quantitatively describe the distribution and composition of species abundance within the communities. As depicted in Figure 1B, the horizontal axis of the curve spanned a wide range, and the curve on the vertical axis was flat, indicating that the operational taxonomic units (OTUs) in our samples were highly diverse and exhibited homogeneous species composition. This richness and uniformity suggest that the sequencing depth was sufficient to capture a broad array of species. To further evaluate sequencing adequacy, a rarefaction curve was generated by randomly sampling data from each sample, which plateaued, confirming that the sequencing data were both sufficient and representative (Figure 1C). Additionally, the box plot demonstrated a tendency to plateau with increasing samples, further suggesting that the sample size was adequate (Figure 1D).

### 3.2 α-diversity

**Figure 2:**
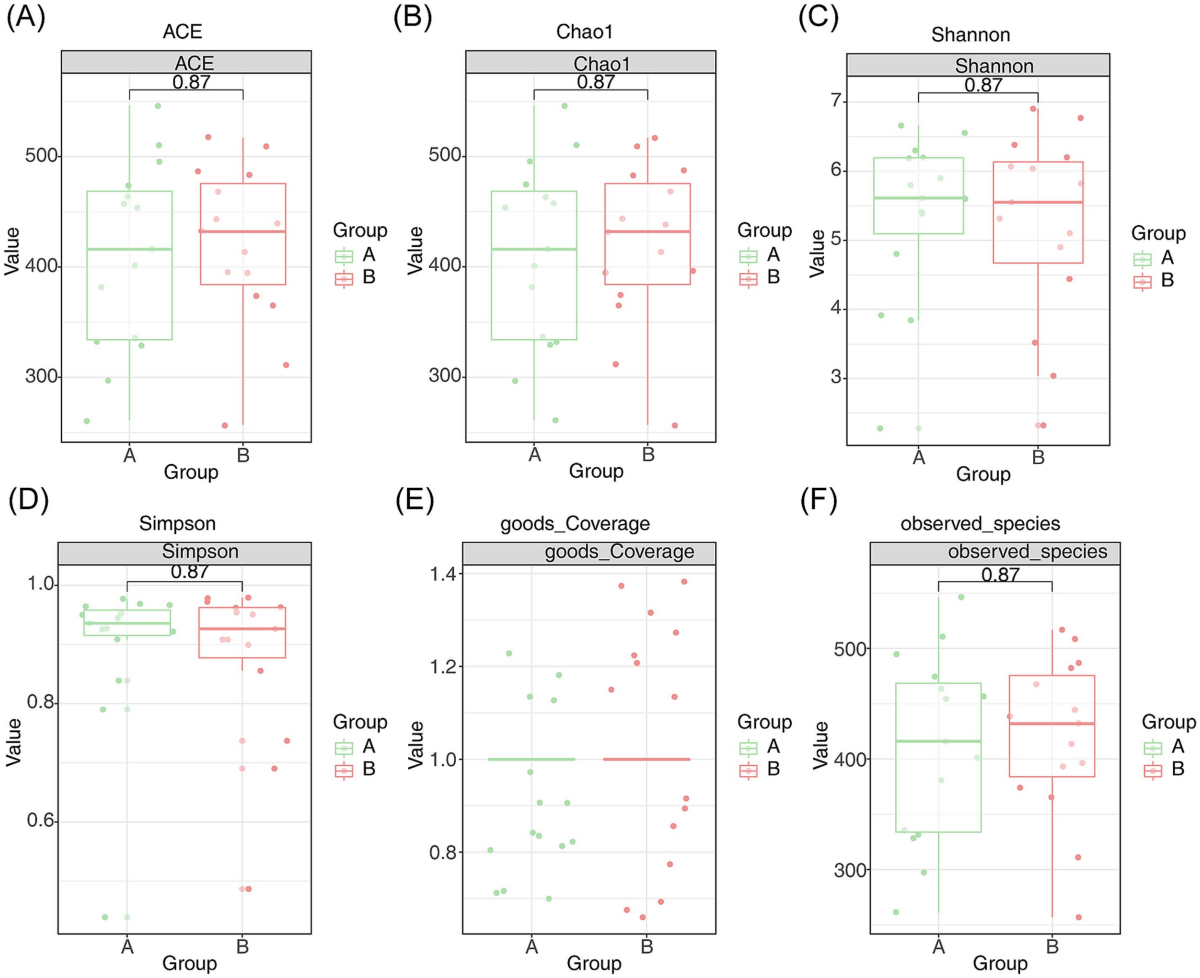
Species diversity analysis of intestinal microcolonies in patients with AR. **(A)** Species richness index, **(B)** ACE index, **(C)** Chao1 index, **(D)** Shannon index, **(E)** coverage index, and **(F)** Simpson index of intestinal microcolonies in patients with AR. Endemic species A are shown in green, and endemic species B are shown in red, with *p* < 0.05 indicating statistical significance.

Microbial diversity was assessed using species richness, ACE, Chao1, Shannon, coverage, and Simpson indices. Although these indices did not show significant differences between the AR and HC groups, they indicated substantial species richness within the samples. Notably, Good’s coverage index approached 1, signifying that the sequencing depth was sufficient to capture nearly all species existing in the samples (Figures 2A–F).

### 3.3 β-diversity

**Figure 3:**
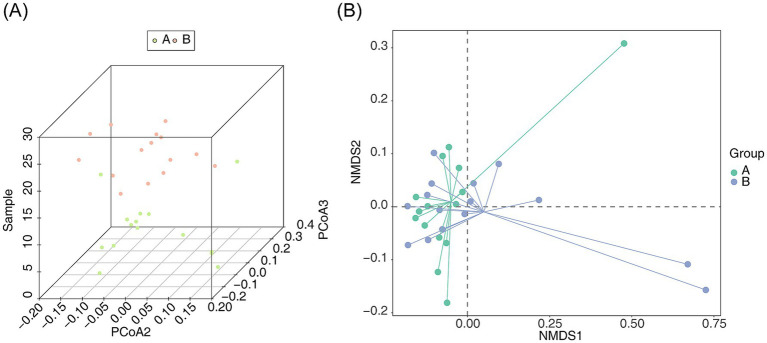
Similarity analysis of bacterial community structure between AR and HC **(A)** PCoA 3D plot and **(B)** NMDS 2D plot for intergroup similarity analysis of bacterial community structure between AR and HC. Points and the distances between them represent the similarity of samples and their bacterial community structures, respectively. Green denotes species endemic to A, and red denotes species endemic to B.

PCoA was utilized to assess the similarity or dissimilarity in community composition between the samples. The 3D PCoA plot separated the microbial communities of the AR and HC groups (Figure 3A). Similarly, NMDS analysis demonstrated distinct clustering between the AR and HC groups (stress = 0.06437842, *p* < 0.05) (Figure 3B), underscoring significant differences in the intestinal microbiota composition between the two groups.

### 3.4 Analyzing horizontal community structure across discrepant groups

**Figure 4:**
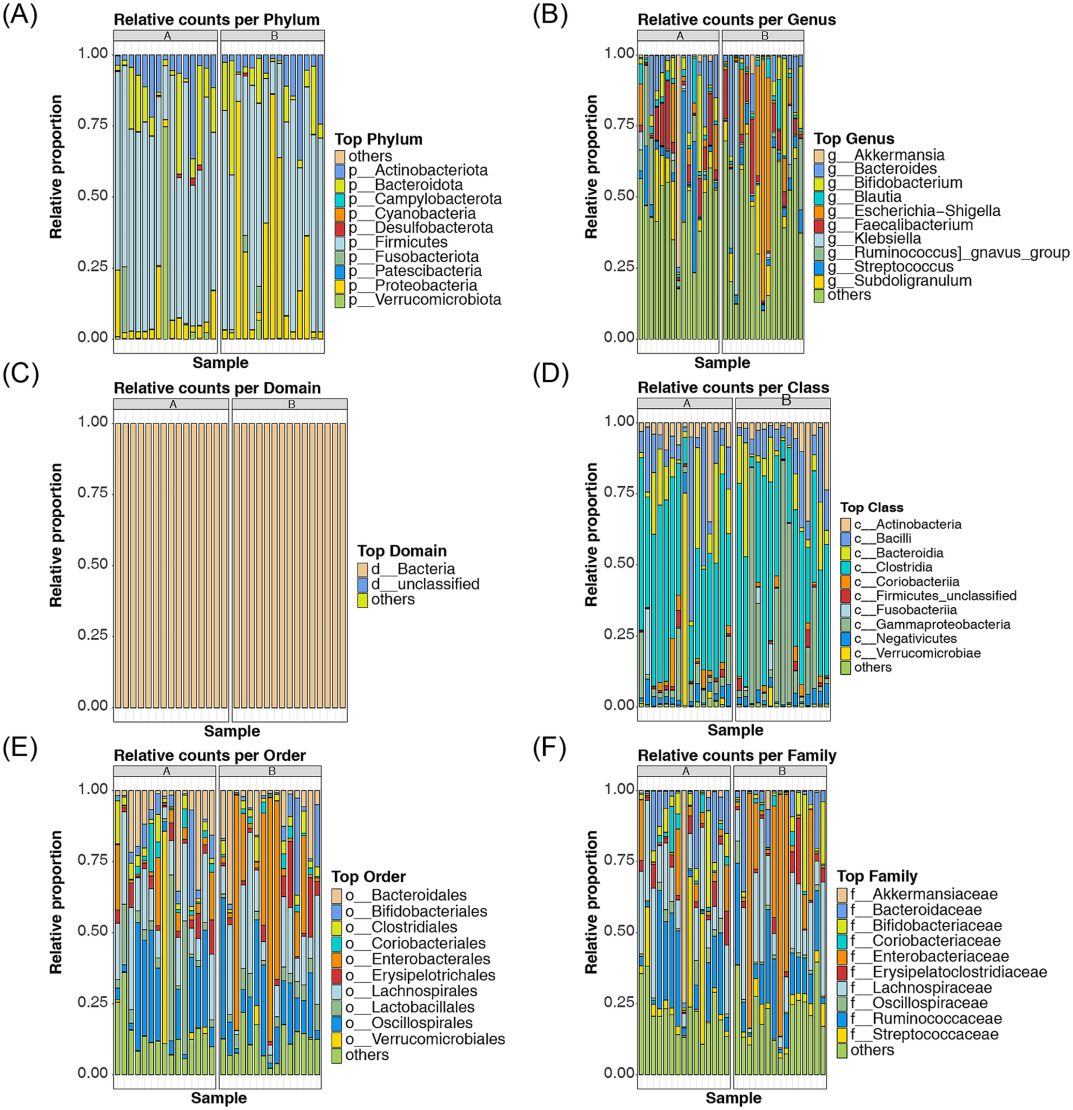
Analysis of the relative proportions of the top 10 most abundant species in AR and HC **(A–F)** The relative proportions of the top 10 most abundant species between AR and HC were analyzed at the phylum, genus, domain, class, order, family, and species levels. Different colors represent distinct phyla, genera, domains, classes, orders, families, and species. Species that are endemic to A are indicated by “a,” and species endemic to B are indicated by “b”.

At the species level, the top 10 most abundant species in both the AR and HC groups were identified, and a cumulative bar chart was generated to depict their relative proportions. At the phylum level, the most abundant taxa included *Actinobacteriota*, *Bacteroidota*, *Campylobacterota*, *Cyanobacteria*, *Desulfobacterota*, *Firmicutes*, *Fusobacteriota*, *Patescibacteria*, *Proteobacteria*, and *Verrucomicrobiota* (Figure 4A). At the genus level, the most abundant genera were *Akkermansia*, *Bacteroides*, *Bifidobacterium*, *Blautia*, *Escherichia-Shigella*, *Faecalibacterium*, *Klebsiella*, *Ruminococcus gnavus group*, *Streptococcus*, and *Subdoligranulum* (Figure 4B). The top 10 taxa at the domain, class, order, family, and species levels are presented in Figures 4C–F; Supplementary Figure S1.

### 3.5 LEfSe analysis

**Figure 5:**
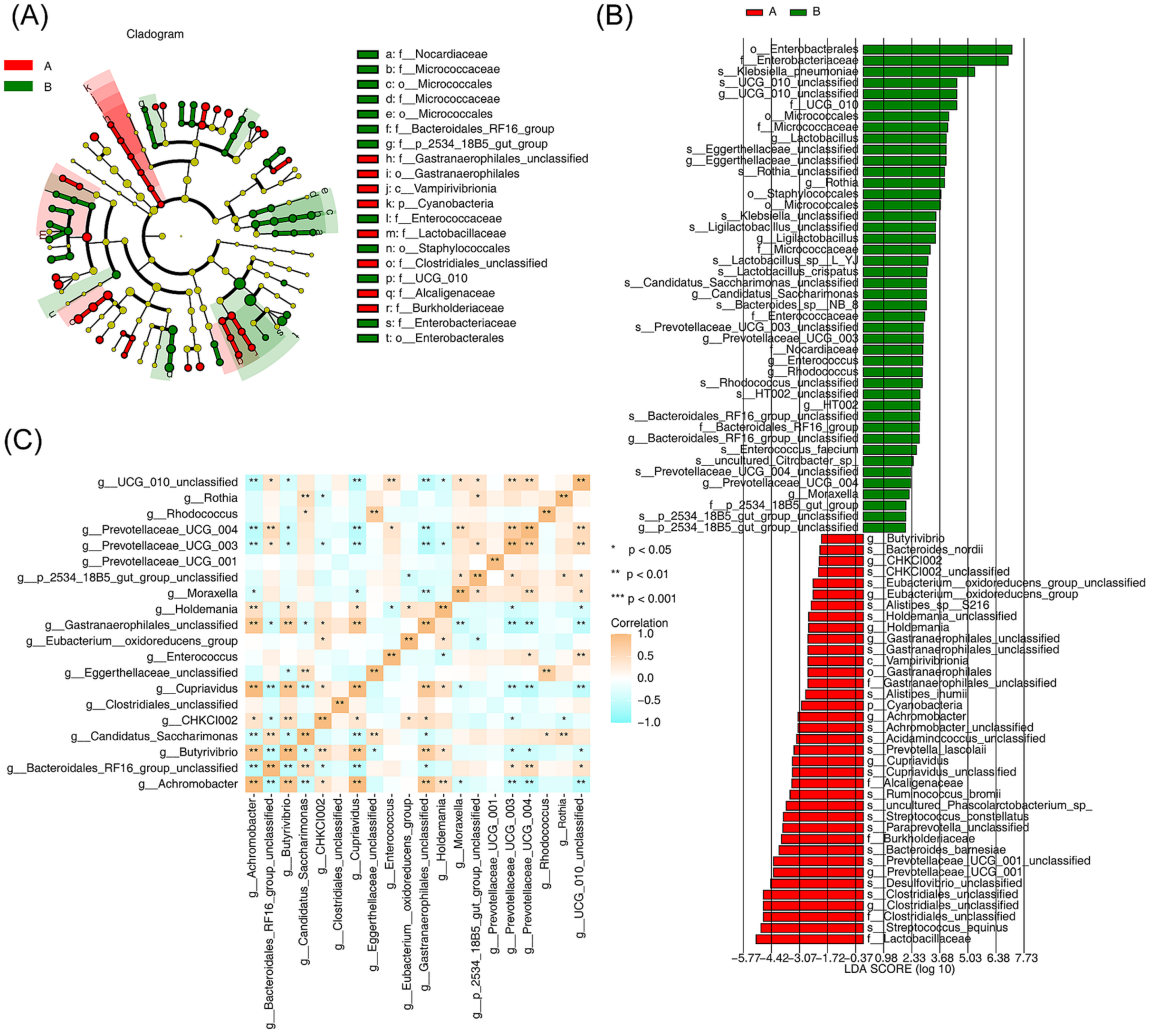
Screening of species differing between AR and HC and analysis of the consistency of their interspecific differences **(A)** Branching diagram analyzing changes in specific bacterial phylotypes between AR and HC. Different letters, dots, connecting lines, and branches represent taxa, their common ancestors, evolutionary relationships, and evolutionary pathways, respectively. **(B)** Screening of significantly different species between AR and HC through LDA analysis. The *x*-axis values represent LDA scores, with red indicating AR-specific species and green indicating HC-specific species (LDA score >4, *p* < 0.05). **(C)** Spearman correlation analysis showing interspecific correlations for species differing between AR and HC. Different colors represent various correlation values, with statistical significance indicated by ^*^p < 0.05, ^**^p < 0.01, and ^***^p < 0.001.

LEfSe analysis was performed to identify specific bacterial phylotypes that differed significantly between the AR and HC groups. The cladogram generated by the LEfSe method (LDA score >4, *p* < 0.05) revealed significant differences in the abundances of 74 bacterial phylotypes between the two groups, with 37 phylotypes being more abundant in the AR group and 37 in the HC group. At the genus level, 20 genera were identified as significantly different, including *Rhodococcus*, *Rothia*, and *CHKCI002* (Figures 5A,B). Spearman correlation analysis further identified significant correlations among eight discrepant genera, including *Prevotellaceae UCG-003*, *Butyrivibrio*, *Prevotellaceae UCG-004*, *Rhodococcus*, *Cupriavidus*, and *Achromobacter*. Notably, *Prevotellaceae UCG-003* exhibited a negative correlation with *Gastranaerophilales* (unclassified), while *Achromobacter* demonstrated a negative association with *Cupriavidus* (*R >* 0.8, *p* < 0.05) (Figure 5C).

### 3.6 A total of seven pivotal micropopulations were identified

**Figure 6:**
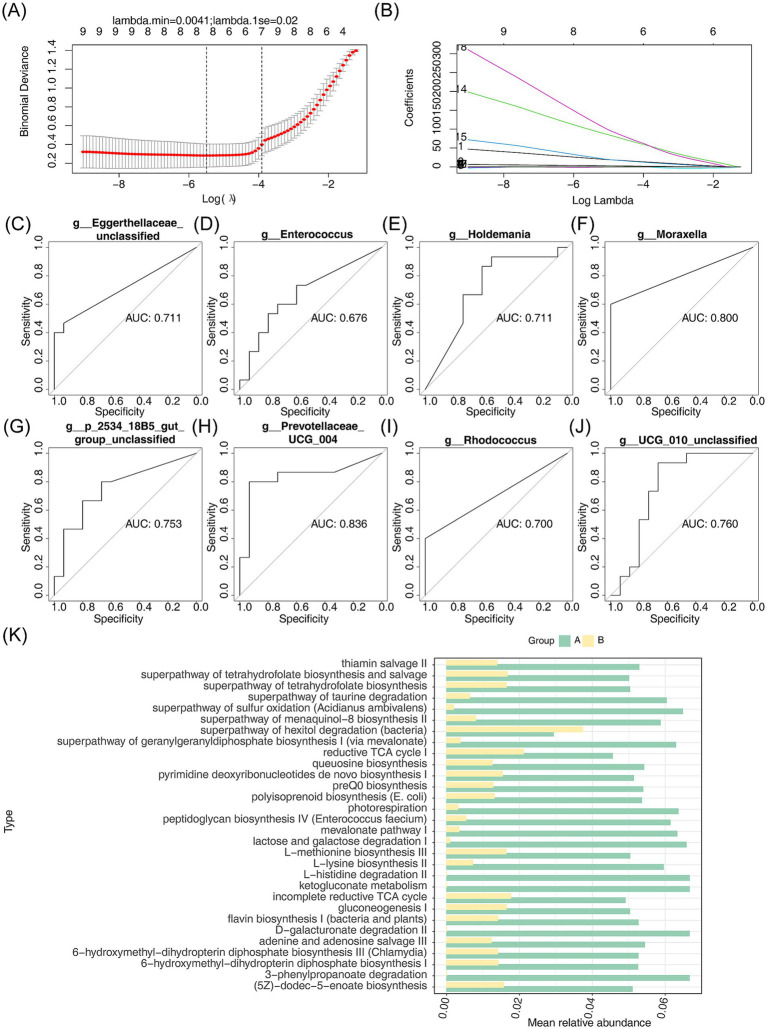
LASSO regression modeling to analyze key microgroups and their enrichment pathways between AR and HC. **(A,B)** Scatterplots and fitted line plots were used to identify key micropopulations between AR and HC. Each point represents a data point, and the slope or shape of the line reflects the relationship between the predictor variable and the response variable. **(C–J)** The diagnostic value of the key microbial groups was evaluated using ROC curve analysis. The area under the curve (AUC) represents the diagnostic performance, with AUC >0.7 indicating good diagnostic value. **(K)** The average relative abundance and enrichment pathways of the eight key micropopulations were analyzed using PICRUSt2. The horizontal and vertical axes represent the average relative abundance and enrichment pathways, respectively.

To identify pivotal micropopulations, the LASSO regression model was applied, resulting in eight key micropopulations (*Rhodococcus*, *Eggerthellaceae* (unclassified), *Enterococcus*, *Moraxella*, *2534 18B5 gut group* (unclassified), and *Prevotellaceae UCG-004*) when the minimum value of lambda was set to 0.0041 (Figures 6A,B). Among these, the AUCs of *Prevotellaceae UCG-004*, *Holdemania*, *UCG-010 unclassified*, *2534 18B5 gut group* (unclassified), *Moraxella*, *Eggerthellaceae* (unclassified), and *Rhodococcus* were all greater than 0.7, qualifying them as pivotal micropopulations with strong diagnostic potential (Figures 6C–J).

Further investigation of the metabolic functions of these pivotal micropopulations revealed significant differences in 209 metabolic pathways. These included pathways related to L-lysine biosynthesis II, the superpathway of geranylgeranyldiphosphate biosynthesis I (via mevalonate), photorespiration, mevalonate pathway I, and the superpathway of sulfur oxidation (*Acidianus ambivalens*) (Figure 6K).

## 4 Discussion

Currently, the diagnosis of AR is based on factors such as symptom frequency and severity, along with skin prick test results. Our 16S rRNA sequencing study revealed significant differences in microbial community composition between healthy individuals and patients with AR. Notably, patients with AR exhibited reduced microbial diversity, accompanied by an increased abundance of specific bacterial taxa, such as *Bacteroidetes*, and a decreased abundance of others, including *Oxalobacter* and *Sutterella* (Watts et al., 2021). Comparative analysis of bacterial composition, abundance, and community structure highlighted statistically significant differences between patients with AR and healthy controls. LEfSe analysis identified 37 bacterial taxa enriched in the AR group and 37 enriched in the healthy group. These shifts in relative abundance suggest that microbiota alterations may play a role in AR pathogenesis. Such findings provide novel insights into AR pathophysiology and emphasize microbial communities’ possible participation in modulating immune responses. Additionally, factors such as diet, environmental exposure, and immune system status likely contribute to these microbial alterations. Dietary patterns can influence gut microbiota composition directly, while exposure to environmental allergens may impact microbial diversity and abundance. Furthermore, early-life bacterial colonization and the host immune response are known to shape immune system development, potentially affecting the onset and progression of AR (Mahajan et al., 2020). These microbial differences are closely associated with the underlying mechanisms of AR pathogenesis.

Species identification results showed that AR and control samples shared 1,122 common species, 1,803 A-endemic species, and 1,739 B-endemic species. This suggests that these species might play a pivotal role in maintaining the stability and functionality of the intestinal micro-ecological environment in AR. Investigating these species could provide valuable insights into their potential roles in AR pathogenesis and management.

The species diversity analysis, including the abundance grade curve, dilution curve, and species accumulation curve, confirmed the reliability of the sequencing data in this study. Estimating terrestrial biodiversity through extrapolation is recognized as an effective method for assessing biodiversity. This approach allows for rapid and approximate evaluations of species richness and faunal or floral composition at comparative sites (Colwell and Coddington, 1994). Standardizing samples based on completeness rather than size, this method minimizes bias in richness comparisons between communities, requiring less total sampling effort. Additionally, when combined with an adaptive coverage-based stopping rule during sampling, direct comparisons between samples become feasible without the need for rarefaction, thus eliminating the collection of excessive data and preventing wastage (Colwell and Coddington, 1994). The Good’s coverage index, which was close to 1, indicates that the sequencing depth was appropriate, ensuring the coverage of majority of species in the samples. Furthermore, the PCoA and NMDS analyses demonstrated significant differences in the community composition of intestinal flora between the AR and control groups. Thus, the diversity and composition of microbial communities may influence the progression of AR or similar diseases, suggesting that microbial dysbiosis could play a role in the development or exacerbation of AR.

LEfSe has proven effective in detecting differentially abundant features in the human microbiome, particularly in mucosal or aerobic taxa and in mouse models of colitis. Comparative analyses with existing statistical methods and metagenomic techniques consistently show that LEfSe has a lower false positive rate while effectively elucidating the biological mechanisms behind microbial community differences (Segata et al., 2011). LEfSe identified 20 differentially abundant microbiota across various taxonomic levels in this study. The LASSO algorithm selected eight key microbial groups, including *Prevotellaceae UCG-003*, *Butyrivibrio*, *Prevotellaceae UCG-004*, *Rhodococcus*, *Cupriavidus*, and *Achromobacter*. Notably, *Rhodococcus* is known to harbor genes involved in lipid metabolism, which are critical for its survival and persistence in diverse environments (Alvarez et al., 2021). Additionally, *Prevotellaceae UCG-004* has been linked to enhanced metabolism of ascorbic acid and uronic acid, potentially increasing antioxidant capacity (Li C. et al., 2022) and influencing AR development. Moreover, the ROC curve analysis demonstrated that these eight key microbial groups have diagnostic potential for AR, highlighting their importance in both the development and diagnosis of the disease.

PICRUSt2 enhances the precision and versatility of inferring marker gene metagenomes (Noecker et al., 2016) by employing an extended ancestral-state reconstruction algorithm to predict gene families, integrating them to estimate the composite metagenome. Utilizing 16S rRNA data, PICRUSt2 recapitulates key findings from the Human Microbiome Project and accurately predicts gene family abundance in both host-associated and environmental communities, with quantifiable uncertainty (Langille et al., 2013). Analysis with PICRUSt2 revealed significant functional differences in gut microbiota between AR and control groups, highlighting the regulatory roles of seven key microbial taxa in the gut microbiota of patients with AR. While direct evidence linking specific bacteria to AR symptom promotion remains lacking, studies suggest that *Streptococcus sali*var*ius* may contribute to AR development, possibly playing a role in its pathogenesis (Miao et al., 2023). Thus, further investigation is needed to elucidate the mechanisms and interactions between specific bacteria and AR.

This study, however, presents several limitations. First, the sample size is relatively small, with an underrepresentation of young patients, which increases the risk of sampling bias. As a result, observed microbial community differences may be incidental and not representative of population-level characteristics. Second, variations in sampling time, methods, and concurrent atopic conditions could influence microbiota composition. These limitations may lead to an overly optimistic interpretation of causal relationships between microbial community differences and AR. To overcome these limitations, future research should involve a larger, more diverse sample with a better representation of younger patients. Additionally, strict control over patient selection criteria, sampling times, and methodologies is crucial for a more comprehensive understanding of the microbiome’s role in AR.

## 5 Conclusion

This study analyzed the gut microbiota of patients with AR, revealing a distinct microbial profile specific to this condition. These microbial communities may play a pivotal role in AR progression, offering new insights into the gut health of patients with AR and enhancing the understanding of the relationship between gut microbiota and overall health status in individuals with AR. Although key microbial communities have been identified, the precise mechanisms and interactions between specific bacterial taxa and AR remain unclear. Further investigation into these interactions will deepen our understanding of the complex mechanisms underlying AR and provide valuable directions for future research.

## Data Availability

The datasets presented in this study can be found in online repositories. The names of the repository/repositories and accession number(s) can be found in the article/Supplementary material.
