# Supplementary material for: 16S rRNA sequencing-based evaluation of the protective effects of key gut microbiota on inhaled allergen-induced allergic rhinitis
Source: Front Microbiol. 2025 Jan 9;15:1497262. doi: 10.3389/fmicb.2024.1497262 (PMC11756352; doi:10.3389/fmicb.2024.1497262)
Supplement: Supplementary file 1 [file Data_Sheet_1.PDF]

Supplementary Material

1.1 Supplementary Figures

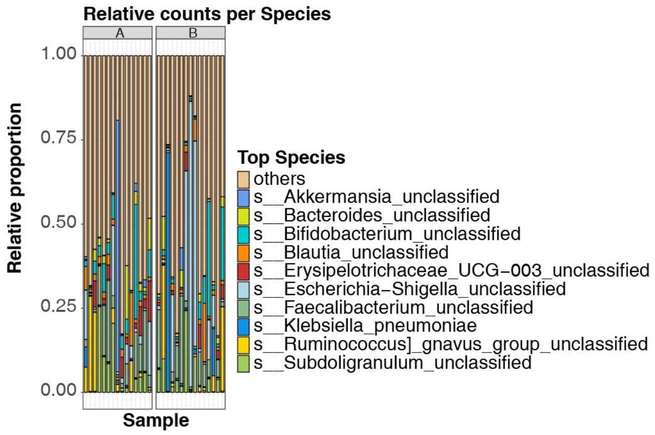

Supplementary Figure 1. The relative proportions of the 10 most abundant species species between AR and HC were analysed at the level of species.
